# Agreements and Disagreements Between Professionals and Users About the Experience of a Telehealth Service for HIV Pre-Exposure Prophylaxis (TelePrEP): Qualitative Interview Study

**DOI:** 10.2196/67445

**Published:** 2025-04-02

**Authors:** Lorruan Alves dos Santos, Luiz Fábio Alves de Deus, Ramiro Fernandez Unsain, Andrea Fachel Leal, Alexandre Grangeiro, Marcia Thereza Couto

**Affiliations:** 1 Departamento de Medicina Preventiva Faculdade de Medicina FMUSP, Universidade de São Paulo São Paulo Brazil; 2 Associação Brasileira Interdisciplinar de AIDS (ABIA) Rio de Janeiro Brazil; 3 Department of Sociology, Humanities and Philosophy Institute, Universidade Federal do Rio Grande do Sul Porto Alegre Brazil

**Keywords:** pre-exposure prophylaxis, HIV, telemedicine, men who have sex with men, health personnel

## Abstract

**Background:**

Men who have sex with men have a disproportionately high prevalence of HIV worldwide. In Brazil, men who have sex with men account for over 15% of HIV cases, substantially higher than the general population prevalence of 0.6%. Pre-exposure prophylaxis (PrEP) is a critical biomedical strategy for reducing HIV transmission, yet adherence remains challenging due to stigma, logistical barriers, and the need for regular clinical follow-ups. TelePrEP, a telehealth-based approach to PrEP follow-up, has emerged as a potential solution to improve accessibility and reduce stigma. However, the perspectives of users and health care providers on this intervention remain understudied in low- and middle-income countries, such as Brazil.

**Objective:**

This study aims to examine the experiences and perceptions of users and health care professionals regarding TelePrEP, an asynchronous remote consultation model, in 5 PrEP services across 3 Brazilian regions (southeast, south, and northeast).

**Methods:**

We conducted 19 in-depth interviews with PrEP users (aged between 23 and 58 years) and 6 interviews with health care professionals (aged between 35 and 61 years). Users were recruited from 5 public health care services, including outpatient HIV clinics and testing centers. The interviews explored motivations for PrEP use, experiences with in-person and remote consultations, perceived advantages and disadvantages of TelePrEP, and overall satisfaction. Thematic analysis was conducted using NVivo software.

**Results:**

Users reported greater convenience, increased autonomy, and reduced stigma, highlighting that the remote consultations eliminated the discomfort of discussing personal topics in person and minimized the need for frequent visits to health care facilities. Many felt that TelePrEP simplified HIV prevention, normalized PrEP use, and contributed to more sustainable adherence while also expressing confidence that periodic laboratory testing was sufficient for monitoring their health. Conversely, health care professionals raised concerns about the loss of personal connection with users, which they perceived as essential for detecting health issues and ensuring PrEP adherence. They also noted that TelePrEP could hinder the identification of sexually transmitted infections due to the absence of direct clinical assessments, and some questioned whether TelePrEP compromised the quality of care, fearing that users might delay reporting symptoms or other health concerns.

**Conclusions:**

To effectively address the needs of both groups, the successful implementation of telehealth PrEP services must consider these differing perceptions. Further research is essential to explore implementation in diverse settings and enhance the training of health care professionals to address the specific requirements of PrEP care.

## Introduction

### Barriers to Pre-Exposure Prophylaxis Effectiveness

Men who have sex with men (MSM) face considerable disparities in HIV prevalence worldwide compared to the general population [1,2]. In Brazil, studies indicate an overall prevalence of 0.6%, while among MSM, the figure is over 15% [3,4]. To address these disproportionate rates, biomedical prevention strategies, such as postexposure prophylaxis and pre-exposure prophylaxis (PrEP), have proven to be essential for interrupting the chain of HIV transmission and are crucial resources for achieving the desired end of the epidemic [5-8]. However, for these approaches to be effective in reducing HIV incidence among the most affected groups, such as MSM, and especially in the case of PrEP, its coverage needs to be increased. For example, mathematical models estimate that 50% of the MSM at substantial risk of HIV infection need to use PrEP for it to have a significant impact on the hypothetical end of the HIV epidemic [9].

In addition to increasing PrEP uptake, maintaining proper use and adherence is a major challenge in the context of combined HIV prevention. Inadequate use and discontinuation of PrEP are complex phenomena that undermine its effectiveness as both an individual and collective strategy. Numerous factors are related to discontinuation of PrEP use, such as substance use [10], low or changed perception of risk [10-13], change in relationship status and sexual partners’ understanding of PrEP [10,13-15], side effects of PrEP [11,13,14], drug intake and fatigue [12], stigma due to the assumption that PrEP users test positive for HIV or have sex with multiple partners [16,17], the need to maintain confidentiality [18], among others.

The recent World Health Organization guidelines [19] recommend reducing the frequency of kidney function monitoring for low-risk individuals, simplifying PrEP follow-up protocols. This recommendation is based on updated safety evidence and aims to minimize unnecessary clinical demands. In Brazil, where logistical barriers often disrupt adherence to PrEP, implementing this approach could address these challenges. By integrating less frequent monitoring into TelePrEP services, care continuity could be improved, aligning with global initiatives to enhance PrEP accessibility and adherence.

Besides these factors, studies indicate that discontinuing PrEP use is strongly associated with difficulties reconciling periodic clinical monitoring with the responsibilities and obligations of daily life. Furthermore, the social stigma related to attending specialized HIV services remains a significant impediment for many individuals. Continuous use of PrEP requires periodic clinical and laboratory monitoring, and evidence indicates that intense work routines, frequent travel, and other personal priorities contribute to the discontinuation of prophylaxis use [20,21]. In addition, the quarterly follow-up required for PrEP renewal is often perceived as burdensome, particularly for individuals seeking confidentiality about their sexual life. This can lead to concerns about exposure and stigma, further hindering adherence [22,23].

### Telemedicine Improving PrEP

To address the difficulties of reconciling quarterly in-person follow-up with work and study routines as well as the stigma associated with visiting HIV prevention services and other barriers that often result in discontinuation of PrEP use, digital health interventions are recommended [24]. Given these barriers, telemedicine emerges as a pivotal tool to address the challenges of adherence and accessibility. Providing follow-up through telemedicine technologies has been identified as an effective alternative to mitigate barriers to accessing PrEP dispensing services and, consequently, ensuring the continuity of prevention. Telemedicine can solve issues related to geographical difficulties, restricted opening hours of health services, and stigmas associated with visiting specialized HIV and AIDS services, as observed in the care of people living with HIV [25]. In general terms, *telemedicine* is defined as the provision of partial or comprehensive health services mediated by telecommunication, including telephone and internet contacts [26].

In HIV prevention, the discussion about the use of telemedicine across the PrEP care continuum predates the COVID-19 pandemic [27]. However, with the outbreak of the pandemic caused by the coronavirus and the imposition of policies to control and restrict movement, there has been an acceleration in the introduction of telemedicine in sexual health care [28,29]. Specifically, in the case of PrEP, adopting telemedicine resources, such as telemedicine consultations and support for self-testing, has shown positive results in promoting access and adherence to PrEP [24]. In Brazil, telemedicine programs involving individuals in PrEP care are rare. However, prompted by the COVID-19 pandemic, some PrEP research projects have adapted their protocols to continue recruiting and providing care through remote monitoring technologies due to restrictions on in-person contact [30,31].

Telehealth has emerged as a promising strategy to improve access and adherence to PrEP globally. Initiatives in high-income countries, such as Iowa TelePrEP [32,33] in the United States, have demonstrated the feasibility of telehealth for expanding geographic coverage and reducing stigma. It is important to highlight that Brazil is a middle-income country [34], and its public health care system faces different structural and socioeconomic challenges compared to high-income settings. Understanding TelePrEP’s implementation in this context can provide valuable insights for scaling up digital health interventions in low- and middle-income countries. In Brazil, the Combine! Study showed that telehealth can reduce discontinuation rates and optimize service delivery by addressing barriers, such as service congestion and travel constraints, aligning with global findings on its potential to enhance HIV prevention outcomes [35].

Innovative strategies have been proposed to improve adherence and retention in PrEP use, including digital interventions, such as TelePrEP, and new methods of administration, such as long-acting PrEP [36]. The latter, administered through periodic injections, has emerged as a promising alternative to overcome barriers related to adherence. However, in Brazil, the availability of long-acting PrEP is still limited to clinical and demonstration studies, with no forecast for implementation in the Brazilian Unified Health System.

Although the COVID-19 emergency has enabled the inclusion of telemedicine in the context of PrEP, issues related to its implementation; effectiveness; and how technology adoption is perceived by users, health professionals, and managers remain little studied. In this context, this study aims to explore and analyze the perceptions and experiences of users and health care providers concerning a modality of clinical monitoring of PrEP with asynchronous remote assessments (TelePrEP) from 5 services with different types of organization and location regions. This analysis aims to inform the development of TelePrEP protocols that are both scalable and adaptable to diverse regional and social contexts in low- and middle-income countries.

## Methods

### Overview

We collected the data analyzed in this study in the third phase of the Combine! Study [35], a longitudinal demonstration study conducted between 2015 and 2023 in 5 cities across 3 regions of Brazil. In the south and southeast regions, the study included 2 HIV outpatient clinics located in Porto Alegre, Rio Grande do Sul and Ribeirão Preto, São Paulo, as well as 2 counseling and testing centers in Curitiba, Paraná and São Paulo, São Paulo. In the northeast region, the study encompassed an infectious disease hospital in Fortaleza, Ceará. The study aimed to evaluate the effectiveness of HIV PrEP and combined prevention strategies in the context of various public sexual health services within the Brazilian Unified Health System.

This is the third phase of the Combine! Study that used mixed methods to investigate the impact of adopting remote and asynchronous clinical follow-up (TelePrEP) on PrEP retention and use as well as its potential effect on the diagnosis of sexually transmitted infections (STIs) and HIV. The TelePrEP protocol involved quarterly asynchronous clinical assessments conducted through an web-based platform, which was accessible to users and health professionals via devices, such as cell phones, tablets, or computers. The TelePrEP protocol included an annual in-person appointment. At the beginning of this third phase, carried out between July 2019 and December 2020, all participants in PrEP follow-up in the Combine! Study for >6 months and those who had regular access to the internet were invited to continue clinical follow-up via remote modality, if desired [35]. In the qualitative component, users and professionals, including health care professionals, administrators, data specialists, working in the Combine! Study sites were invited to participate.

### Interview Procedures

For this paper, we analyzed qualitative empirical data collected from users identified as cisgender men who were or had experience with PrEP follow-up in remote and asynchronous mode, as from health professionals responsible for the remote or in-person clinical follow-up of users, most of whom were infectious disease specialists from all the Combine! Study sites.

Potential users were preselected based on medical records, sociobehavioral questionnaires from the study, and criteria ensuring the expected sample diversity. Users were invited to participate via telecommunication apps by field researchers. Those who accepted were referred to by the qualitative research team that provided a detailed explanation of the study’s objectives and procedures before obtaining informed consent. Health care professionals, in turn, were selected based on their involvement in the TelePrEP component of the study. All professionals engaged in TelePrEP were invited to participate in the interviews. This approach ensured the inclusion of key stakeholders in TelePrEP implementation, allowing a comprehensive understanding of different perspectives on the service.

A total of 19 users and 6 health care professionals were interviewed as part of this study. This number of interviewees was determined by the theoretical saturation criterion [37,38]. This methodological criterion, widely used in qualitative research, guides the cessation of data collection when sufficient thematic recurrence is observed to address the research question, often accompanied by the absence of new elements or relevant information in participants’ narratives. In this study, saturation was confirmed by the authors after reviewing the interviews that were conducted. Among the prescribing professionals, all those who agreed to participate in the research were included, and due to the limited number of health care professionals in the participating services, diversity could only be ensured in terms of age and race categories.

The user interview script (Multimedia Appendix 1) included sociodemographic questions, motivations for using PrEP, and experiences with in-person clinical follow-up and TelePrEP. In addition to sociodemographic questions, the interview script for professionals (included in Multimedia Appendix 1) investigated their professional trajectory; work with PrEP, including their regular interactions and procedures with PrEP users; and experiences with in-person clinical follow-up and TelePrEP. The scripts were tested in a pilot phase. In the user group, we sought to ensure diversity across age groups; race or color; time of PrEP use; and health services that include specialized clinics, testing centers, and hospitals.

The interviews were conducted by trained researchers with previous experience in qualitative research and HIV prevention. All interviews, except those with health care professionals prescribing PrEP from the São Paulo service, were conducted virtually between May and June 2021 due to restrictions imposed by the COVID-19 pandemic. All in-depth interviews were conducted via video call platform and recorded after participants gave explicit consent and signed an informed consent form. Code names were assigned to participants to maintain confidentiality. The material was then transcribed and reviewed, and a thematic analysis was conducted using NVivo software (Lumivero).

### Analysis Strategy

The data were analyzed and interpreted using thematic analysis [39], focusing on the content and contextual meaning of the narratives of users and health professionals. We followed the following steps in the inductive analytical-interpretative trajectory of the empirical material: (1) comprehensive reading, aiming at the impregnation, overview, and apprehension of the particularities of the transcribed interviews; (2) identification and thematic cut that emerges from the narratives; (3) identification of patterns of explicit and implicit meanings in the statements, carried out by 3 researchers, one responsible for conducting the interviews and 2 others as verification specialists; (4) search for broader (sociocultural) meanings underlying the statements of the research participants; (5) dialogue between the problematized ideas and the comparison with the literature; and (6) work of dialogic elaboration among the researchers of an interpretative synthesis, based on the objectives of the study, the results, and the discussions produced from the literature on the theme or object.

### Ethical Considerations

All requirements established by resolutions 466/2012 and 510/2016 of the National Health and Ethics Councils were fulfilled, and the study was approved by the ethics committee of the University of São Paulo Medical School (approval number 3.438.329/2019) before the commencement of field research. Written informed consent was obtained from all study participants. Privacy and confidentiality of the data were ensured, with all data anonymized or deidentified for analysis by the study coordination team. Users received a stipend of R$ 40 (approximately US $7 at the exchange rate of July 23, 2024) to cover any expenses related to their participation in the qualitative component of the study. Participants were assigned pseudonyms to prevent identification, and no image recording was required.

In addition, this study incorporated measures to address researcher positionality and mitigate power dynamics with participants. Researchers underwent training to ensure cultural sensitivity and neutrality during interviews. The interview environment was designed to be welcoming and nonjudgmental, empowering participants to share their experiences openly. Moreover, the researchers conducting the interviews had extensive experience in working with sexual, gender, and racial minorities, ensuring a nuanced and empathetic approach to the sensitive nature of the study.

## Results

### Participant Profile

Of the 19 users using TelePrEP, 17 (89%) had completed higher education, while 2 (11%) had started but not completed higher education. All (n=19, 100%) users identified as cisgender men and were aged between 23 and 58 years. In addition, 12 (63%) users identified themselves as White and 7 (37%) as Black (Black and mixed race combined), a characterization consistent with the PrEP users’ profile in Brazil and the cities where the study was conducted [40,41]. Of these respondents, 3 (16%) preferred to return to in-person PrEP follow-up at some point during their study participation, and 1 (5%) discontinued PrEP use at baseline (>60 days without PrEP) but remained on TelePrEP after being offered (Loki).

Among the 6 professionals prescribing PrEP, 4 (67%) were women and 2 (33%) were men, all of whom identified as cisgender and were aged between 35 and 61 years. Some (n=4, 67%) professionals self-identified as White, while 2 (33%) identified as mixed race (ie, “pardo”).

All women (4/4, 100%) identified as heterosexual, while all men (2/2, 100%) identified as homosexual. Moreover, 83% (5/6) of professionals reported affiliation with a Christian-based religion. Detailed sociodemographic information of the participants is available in Tables 1 and 2.

Most (5/6, 83%) of the professionals interviewed were prescribing physicians, and 1 (17%) was a nurse who managed the health service. In the description of their academic and professional careers, despite specializing in infectious diseases, in the case of physicians, all professionals reported that working in HIV prevention was not an option considered or planned at the beginning of their careers but rather the result of coincidences, such as a coordinator position opening or a new sexual health clinic that needed prescribers.

**Table 1:** Characterization of TelePrEP users.

Fictitious name	Age (y)	Race or skin color	Gender identity and sexual orientation	Education	Religion or spirituality	Type of follow-up at the time of the interview	Research site
Adônis	37	White	Cisgender homosexual man	Completed higher education	Christian	TelePrEP	Porto Alegre, Rio Grande do Sul
Agamenon	33	White	Cisgender homosexual man	Completed higher education	Christian	TelePrEP	Ribeirão Preto, São Paulo
Amon	27	White	Cisgender homosexual man	Completed higher education	Practices spirituality	Tried TelePrEP but returned to in-person follow-up	Ribeirão Preto, São Paulo
Dionísio	29	White	Cisgender homosexual man	Completed higher education	Wicca	TelePrEP	Fortaleza, Ceará
Édipo	23	Black	Cisgender homosexual man	Completed higher education	Umbanda follower	TelePrEP	Curitiba, Paraná
Érico	30	White	Cisgender homosexual man	Completed higher education	Atheist or agnostic	TelePrEP	São Paulo, São Paulo
Eros	31	Black	Cisgender homosexual man	Completed higher education	Atheist or agnostic	TelePrEP	Curitiba, Paraná
Ícaro	58	White	Cisgender homosexual man	Completed higher education	Umbanda follower	TelePrEP	Curitiba, Paraná
Kael	25	Mixed race	Cisgender homosexual man	Completed higher education	Atheist or agnostic	TelePrEP	Curitiba, Paraná
Lázaro	30	Black	Cisgender homosexual man	Completed higher education	Christian	TelePrEP	Fortaleza, Ceará
Loki	40	White	Cisgender homosexual man	Completed higher education	Lutheran	Stopped using PrEP^a^	São Paulo, São Paulo
Merlin	35	White	Cisgender homosexual man	Completed higher education	Polytheist	TelePrEP	São Paulo, São Paulo
Odin	24	Mixed race	Cisgender homosexual man	Completed higher education	Christian	TelePrEP	Porto Alegre, Rio Grande do Sul
Ran	35	White	Cisgender homosexual man	Completed higher education	Mystic	Tried TelePrEP but returned to in-person follow-up	São Paulo, São Paulo
Thor	33	White	Cisgender homosexual man	Completed higher education	Christian	TelePrEP	Curitiba, Paraná
Tyr	39	Mixed-race	Cisgender homosexual man	Completed higher education	Spiritist	TelePrEP	São Paulo, São Paulo
Uller	28	White	Cisgender homosexual man	Completed higher education	Spiritist	TelePrEP	São Paulo, São Paulo
Aud	44	White	Cisgender homosexual man	Incomplete higher education	Spiritist or Buddhist	Tried TelePrEP but returned to in-person follow-up	Porto Alegre, Rio Grande do Sul
Lino	39	Mixed race	Cisgender homosexual man	Incomplete higher education	Rosicrucian order	TelePrEP	Ribeirão Preto, São Paulo

^a^PrEP: HIV pre-exposure prophylaxis.

**Table 2:** Characterization of TelePrEP health care professionals.

Fictitious name	Age (y)	Race or skin color	Gender identity and sexual orientation	Education	Religion or spirituality	Research site
Asclépio	61	White	Cisgender homosexual man	Completed higher education	No information	São Paulo, São Paulo
Galeno	49	Mixed race	Cisgender homosexual man	Completed higher education	Spiritist	São Paulo, São Paulo
Anahit	38	White	Cisgender heterosexual woman	Completed higher education	Lutheran	Curitiba, Paraná
Cardea	41	Mixed race	Cisgender heterosexual woman	Completed higher education	Christian	Fortaleza, Ceará
Higéia	51	White	Cisgender heterosexual woman	Completed higher education	Spiritist	São Paulo, São Paulo
Panacéia	35	White	Cisgender heterosexual woman	Completed higher education	Lutheran	Porto Alegre, Rio Grande do Sul

### Perspectives and Motivations for Choosing TelePrEP Among Users

According to the testimonies, the main reasons for migrating from traditional PrEP to TelePrEP were the gains in terms of convenience, practicality, and protagonism in sexual health care and HIV prevention because TelePrEP made it possible to reconcile the demands of PrEP with the needs of personal and professional life. Kael highlighted that TelePrEP reduced the stress caused by the stigma related to HIV services, in addition to gains in practicality and autonomy in the care process:

...I took a step further in my prevention. Moreover, man, if it becomes more accessible and more practical, wow, even better for me. But I think about other people too, because I’m not ashamed to go there, but I know that many people are.

Kael; mixed race participant aged 25 years

For other interviewees, TelePrEP minimized problems commonly faced at each consultation at the service, as explained by Eros:

...it was tough, so much so that there were times when I didn’t take [the medication], and I stopped taking it because it was really hard to do all this stuff. After all, you have to wait for the exam, which, let’s say, is 30 minutes, but it went on for almost an hour. Then, you had to hand the exam over to the doctor and wait for the doctor’s time, which went on for about an hour and a half. Then, there was the line to get the medication, which was always very crowded. Even when it was empty, sometimes it took a long time.

Eros; Black participant aged 31 years

Most users positively evaluated the in-person care service professionals (such as physicians, receptionists, and counselors) provided. However, there were also significant reports of embarrassment due to the content of the questions asked by the professional when filling out the PrEP prescription forms and medical records. In addition, some users felt uncomfortable answering protocol questions, which, in the opinion of at least 1 participant, the professional already knew the answer to, especially questions regarding sexual behaviors and practices:

Look, the only thing that changes [in telePrEP] is about embarrassment, perhaps. I’m pretty calm, but whether I like it or not, there are very personal questions that I think I felt more embarrassed about [in face-to-face clinical consultations] when she asked me because she knew the answer, and I had to answer, you know?

Lázaro; Black participant aged 30 years

Along the same lines, another perceived advantage of TelePrEP was the attenuation of the anticipated stigma associated with HIV, as the interviewee would no longer be seen by third parties (ie, sexual partners, friends, or family) attending health services that serve people living with HIV or AIDS or would no longer have to present a medical certificate from these services to justify absence from work. Adônis stated this as follows:

I’ve already gotten there, and there were people I had already seen on the street or something like that. Then the person looks at you, with eyes...Then you always wonder: is he using PrEP, too? Or is he here because he has HIV?

Adônis; White participant aged 37 years

In another statement, Thor explained as follows:

Even today, I feel a little embarrassed; it could be just my imagination because it’s the same place where people get [collect] medication for HIV.

Thor; White participant aged 33 years

It is essential to highlight that the PrEP vials prescribed in TelePrEP should be collected in person at the services' pharmacies during the TelePrEP protocol. In other words, users were required to visit the health care facility, wait for assistance from the pharmacist, and then receive their new medication bottles. This reflected the current stage of TelePrEP implementation in Brazil, where home delivery of medications is not yet a standard practice. However, the statements analyzed indicated that the time spent in and traveling to health services was considerably reduced compared to entirely in-person clinical follow-up:

So the advantage of doing it remotely was that it made things way more practical, right? I basically only had to go there to pick up the medication. I didn’t have to go there twice...Normally, in one week, I’d spend at least two hours there to get all the tests done, you know? It’s quick—I don’t think two hours is a lot of time at all; I actually think it’s really fast. I get there, check in, fill out the form, do the tests, and that’s it—I’m out.

Ran; White participant aged 35 years

Some interviewees also considered that there was no need to carry out in-person clinical consultations for the use of PrEP due to a perception of low risk of adverse effects associated with the medication and the perception that routine laboratory tests alone would be sufficient to assess physical health. Thus, these tests, in themselves, generated a higher perception of safety:

Of course, I know that any medication has side effects. But since we do the exams every three months, they take care of everything. I am also sure that even if it were from a distance, that way, if the doctor looked at your exams there, he would call you if necessary, and if it wasn’t required, it’s one less person on the schedule and another person who needs it, who can be seen, you know?

Aud; White participant aged 44 years

The interviewees noted that the PrEP care process could be simplified, expedited, and made more practical. In addition, they perceived TelePrEP as not only reducing costs for users but also providing economic benefits and optimizing work processes for professionals and health services. For instance, TelePrEP could increase the availability of consultations without necessarily compromising the quality of care or the safety of prophylaxis use. Users also emphasized that the absence of in-person consultations would not undermine their health or access to services, as they retained the option to seek care whenever necessary. Kael, Uller, and Érico highlighted these points as follows:

I think it took away the idea that prevention is complicated and complex. I guess it somewhat normalized prevention.

Kael; mixed race participant aged 25 years

I think this system is an efficient, fast way. Like, I save these people’s time, I save mine, you know?...Obviously, maybe one day a doubt will come up, but I also know that if I want to go and see a doctor, I’ll be able to, you know? For me, it’s great; it works well like this.

Uller; White participant aged 28 years

I had syphilis a third time while I was already on telePrEP, and I contacted [professional], and she managed to schedule an appointment for me, and I went there. So, for me, this was just proof of what I already imagined: if I need an appointment at any time, I’ll be able to get it. I won’t be left, I won’t be left without one, in that sense.

Érico; White participant aged 30 years

### Perspectives of Health Care Professionals on the Challenges of PrEP Care

Many professionals who were interviewed highlighted that monitoring users using PrEP presented significant challenges for health professionals who worked in sexual health care and care of the lesbian, gay, bisexual, transgender, queer, intersex, asexual, and other identities population. They pointed out that their academic training did not adequately prepare them to understand and care for people who do not correspond to hegemonic social standards of identity and sexual orientation. These professionals recognized that PrEP monitoring, whether in person or remote, required an understanding and management of the diversity of gender and sexual identities as well as sexual practices to meet the needs and demands of these groups adequately. In the case of PrEP users, sexual practices and behaviors were even more highlighted in the clinical encounter than when caring for patients who tested positive for HIV, which created discomfort and a feeling of unpreparedness in some professionals:

So, I think that PrEP brought all of these [socially stigmatized topics] to the table, let’s say, and these are things that we were not prepared to do. We were unprepared to work with these issues at any point in our training, right? It’s a parallel universe where no subject will teach this, where there is no place to study it.

Galeno; mixed race professional aged 49 years

In relation, like, in my residency training [in infectious diseases], we didn’t have much of this [to socially stigmatized topics, such as sexuality, gender issues, etc.] because the residency itself was more about the ward with few outpatient clinics, and talking about sexuality was more about PrEP, you know?

Cardea; mixed race professional aged 41 years

This perception of “unpreparedness” was reflected in 2 other tensions. The first was how the person on PrEP was perceived in the care relationship. For Galeano, this tension occurs because people on PrEP do not fit into the classic representation of a “patient,” whose professional intervention is directed at promoting a cure or treating an illness or a disease, which is not the case with PrEP care. As highlighted in the statement by Galeano, “First, they were not patients, they were users,” who came to the office with demands on sexual health and interest in new HIV prevention technologies. This problematization about the training and role of the infectious disease specialist was also brought up by the coordinator of one of the PrEP care services, who faced these dilemmas in managing the service. According to the coordinator, she was frequently confronted by infectious disease professionals in the service who refused to act in prescribing PrEP because they understood that the training and duties of the infectious disease specialist, in the context of that service, were related to HIV treatment and that prescribing PrEP was not the professional’s responsibility:

“I am an infectious disease specialist. I treat HIV. I treat people who have the virus. I do not treat people who do not have the virus. So I do not provide PrEP care” [referring to the everyday discourse of professionals who do not want to prescribe PrEP]...So this discussion ends up generating, sometimes, some conflicts, and I have a minimal number of medical professionals who prescribe [PrEP] here [in the health service].

Anahit; White professional aged 38 years

Still, to exemplify the challenge and complexity of caring for a PrEP user, in one of the interview excerpts, Galeano reported on the need to establish and create bonds with users as a fundamental step in the PrEP care process, as illustrated in the following quote:

I know, more or less, a little about their life context, what they do, their profession, how they deal with this issue of sexuality, from the point of view of whether they are more focused on sex or focused on relationships or whether they are always looking for a relationship with intermittent, intercurrent sex...

Galeno; mixed race professional aged 49 years

Professionals valued the bond created through in-person care, which led to significant concerns about TelePrEP, even though it was initially evaluated positively. They acknowledged the potential benefits of asynchronous telehealth care, such as greater convenience, practicality, and agility for users, which could improve adherence and retention of prophylaxis. This duality between the appreciation of the bond, which was established in the face-to-face relationship, and the potential gains of TelePrEP made professionals limit the benefits of TelePrEP care to a portion of the people on PrEP:

Some people won’t adapt to remote care, right? And other people will like it because it’s more practical, easier or faster, right? This will make it easier to adhere to because you have to call, make an appointment, go to the appointment, and wait. It’s a process for a young person who works and lives full of commitment. Sometimes that makes it difficult, right?

Panaceia; White professional aged 35 years

Thus, according to reports from most professionals, choosing TelePrEP was seen as a renunciation of the bond provided by face-to-face care or even prioritization of other benefits, such as convenience and practicality, to reconcile the need for clinical follow-up and PrEP use:

Well, some patients didn’t want to go to telePrEP because they said they didn’t want to lose contact with me. “Doctor, I don’t want to stop seeing you.” Then I said: “Oh, my God, I want to see you too.” Then there are people who: “No. It’s better for me [to switch to telePrEP].” Then, sometimes, we forget the patient’s face.

Cardea; mixed race professional aged 41 years

On the part of health professionals, the fear of losing the connection was accompanied by the concern that TelePrEP would compromise the quality of the care provided. This feeling was reinforced by the perception that telehealth care was something mechanical, provided with less rigor, and that the lack of face-to-face interaction made it difficult for professionals to identify health problems or even prevent people on PrEP from reporting their concerns. It could lead, for example, to underdiagnosis of STIs:

...it becomes a very mechanical thing that leads to loss of quality. You lose quality. I do not doubt that the quality of care is lost.

Galeno; mixed race professional aged 49 years

...need more rigorous monitoring. Because I think that something can get lost in this environment, you know? The patient may have some, I don’t know, some symptoms, and there’s no way to [diagnose it], right? It gives you that feeling, and there’s no way to express it remotely.

Radius; White professional aged 27 years

Some people on PrEP, specifically those who chose to return to face-to-face follow-up, also perceived the connection and resolution of health problems in face-to-face consultations as positive. These narratives reinforced satisfaction with the connection established with professionals in face-to-face consultations and how useful they were for resolving different health demands related or not related to PrEP:

So by going there in person, I can ask questions about whether this medication interferes with PrEP or not, I can have a conversation with the doctor about other issues related to PrEP, and I can also find out about my sexual orientation.

Amon; White participant aged 27 years

## Discussion

### Principal Findings

Overall, our results indicate significant differences in how health care professionals and PrEP users perceived PrEP follow-up based on asynchronous remote care. TelePrEP proved more advantageous for users than in-person follow-up, being perceived as more practical and convenient and offering greater autonomy in the care process. Contrary to the fear reported by health professionals in our research, users were more confident that remote care did not harm the bond between the service and health professionals. In addition, our findings regarding reduced stigma and discrimination and improved access and convenience for telehealth PrEP users converge with the findings of previous studies.

Bonett et al [42] reviewed TelePrEP programs in the United States and highlighted these benefits as essential to increase user satisfaction and access to care. Notably, only 1 study identified a lack of personal interaction with health professionals as a potential drawback reported by some patients. While this issue was less evident in our research, further investigation is warranted given the nascent stage of TelePrEP implementation in Brazil. Our study advances the literature by contextualizing TelePrEP within the Brazilian setting, where digital health technologies for sexual health care and HIV prevention are still in their early phases. By addressing cultural and systemic factors specific to this region, our findings aim to inform the development of strategies that are carefully planned and sensitive to the diverse perspectives of key stakeholders—professionals, managers, and users—within the PrEP care continuum.

In addition, users also highlighted that embarrassment caused by the need to verbally share details of their sexual behaviors and practices with the health care professional in in-person consultations as well as exposure to the stigma of attending specialized HIV services decreased with TelePrEP, making the remote PrEP clinical follow-up modality being perceived as more enjoyable for the continuity of HIV prevention with the use of PrEP.

While the users’ reports regarding TelePrEP were highlighted by the emphasis on the perceived advantages of the in-person modality, the reports of health professionals were marked by the perception that PrEP care via telehealth is affected by the loss or reduction of contact and bond between the health professional and the prophylaxis user. For many professionals, face-to-face consultations allow the creation and strengthening of the bond with the users, which would be necessary to identify demands related to comprehensive care and diagnose possible STIs. The safety in maintaining PrEP care and assistance, the periodic performance of laboratory tests, as well as the availability of the service to meet their health demands, combined with the perception that they could access the service in person, when necessary, were mentioned by users as points that guarantee trust in the new model of clinical follow-up of PrEP. In this sense, users were confident that remote care did not harm the bond between professionals and the quality of care.

From the health professional’s perspective, the experience with TelePrEP was permeated by the combination of aspects that limit the expansion of the effective use of prophylaxis, such as, first, the insufficiencies in medical training to act in the prevention and care of sexual and gender minorities [43-45]; second, the conflicts in understanding the possible degree of autonomy of people who use PrEP [46]; and, third, the requirements of PrEP prescription protocols that can hinder regular clinical follow-up, as already discussed in other studies [47].

However, in addition to increased autonomy, people on PrEP perceived the lack of obligation to go to the service as an opportunity to eliminate the constraints arising from the hegemonic expectation of sexual practices positioned in a sexual hierarchy, as discussed by Rubin [48], and to avoid an expectation of stigma related to HIV [49]. We believe that these 2 reasons are not trivial. They have constituted essential barriers to the use of preventive methods based on the use of antiretrovirals and have even imposed embarrassment on their users [49-51]. So much so that strategies to reduce the negative impact of these events, such as self-administered questionnaires and different teleconsultation systems, have shown benefits in access and the user-service-professional relationship [52,53].

For health care professionals, in turn, the benefits of TelePrEP emerge mainly from a theoretical narrative rather than from a practical perspective. That is because, although there is no ideal and perfect care for professionals in face-to-face consultations, there is a tendency to believe that this modality is more comprehensive and complete than asynchronous telehealth assessments. Furthermore, for these professionals, the choice of remote follow-up, motivated by convenience and practicality, obliterates recognizing the importance of the bond created in face-to-face relationships, valued more by the professional than by the user, and the endogenous loss of quality of care. This perception occurs despite a shared view with users that face-to-face care and its quality are already eroded for several reasons that directly affect professionals and users, such as the large number of appointments, the accumulation of work, the long waiting times for appointments, and the difficulty—for some and the embarrassment for others—in dealing with sexual and identity diversity.

While the asynchronous nature of TelePrEP may help reduce tensions between users and health professionals by minimizing direct confrontation and stigma, it may also lead to unintended consequences, as some providers may feel less motivated to provide the service due to perceived difficulties in maintaining a meaningful connection with certain users. Furthermore, to compensate for the lack of real-time interactions, providers may request more frequent appointments than necessary, potentially increasing the burden on users. These nuances highlight the complexity of implementing TelePrEP and emphasize the need for more research on how best to balance accessibility, autonomy, and quality of care. Thus, a possible improvement in the service and supply of PrEP with the incorporation of TelePrEP, due to the higher rationalization of demand and care, becomes prohibited by a clinical practice that values face-to-face care learned from the beginning of the training.

Furthermore, there is also an understanding that individuals on PrEP in asynchronous telehealth follow-up may have limitations or even an inability to perceive health needs that could potentially impact the path to seeking the service to diagnose and treat health problems. This perception prevailed despite the awareness that the clinical examination in in-person PrEP care is generally performed in the presence of a clinical complaint expressed by the individual or in the presence of changes in laboratory tests. In other words, as already explored in other studies [54,55], a conception prevailed among professionals that virtuality cannot supplant or overcome material reality, so much so that the study [35] carried out in the same services showed that the frequency of STI diagnosis after 2 years of implementing TelePrEP remained similar to that of in-person care, as well as the rates of HIV and adherence to PrEP were the same. However, the PrEP discontinuation risk was reduced by one third of those who chose TelePrEP [35].

It is clear that the search for and provision of care, particularly in the context of PrEP, is not just about physical presence or virtual immateriality. Instead, it concerns the bond between the users and the health professional. This bond, as the users themselves have defined, is built on trust and care. This emphasis on trust underscores its pivotal role in health care relationships and preventive medicine.

In short, the differences in medical practice representations of how to perform PrEP care generated tension between professionals and people using it. Professionals presented a representation that cannot ignore the body materiality of the user in the office, a classic act of medical theatricality learned through professional training processes. People on PrEP, aware of the imponderables that cross them and eager for strategies that mitigate the transits that aim at prevention, constructed a representation that brings them closer to complete autonomy, or self-care, thereby empowering them in their health care journey. It is important to point out that this tension did not obliterate the prevention process as a whole, still permitting PrEP users to continue the medication use.

To address this issue, we will need policies that involve training professionals, those not yet familiar with this practice, to use this technology, educate people on PrEP, and guide professionals to make safer clinical decisions. In this context, it is worth remembering that a large part of the TelePrEP protocol and the interviews analyzed here were conducted during the COVID-19 pandemic in Brazil and that, even so, some prescribing professionals from the services participating in the study were resistant to telehealth.

Training must include new technologies, to better equip the health professionals in providing TelePrEP services, as the professionals’ narratives suggested when they highlighted challenges and insecurities generated by academic and professional deficiencies, which did not qualify them to work in PrEP fully. It is worth recalling that none of the professionals planned to work in HIV or STI prevention, even those with medical specialization in infectious diseases. According to the statements of most professionals, medical training was focused on providing health services for people living with HIV but did not include diversity and inclusion training on dealing with sexual minorities.

Several studies from the United States, the United Kingdom, and Brazil address the lack of academic courses and institutional training on caring for sexual and gender minorities [44,45,50]. Most studies indicate that the lack of certain essential topics in professional training is prevalent in educational institutions worldwide, regardless of their economic status or level of prestige. As a result, professionals educated in these institutions often recognize their shortcomings in providing care to vulnerable populations [44,56,57]. Although the National Curricular Guidelines explicitly state the importance of these themes in medical training to ensure adequate care for people from sexual and gender minorities, studies that investigated the curricular components of Brazilian medical courses also identified the lack or scarcity of themes related to gender, sexuality, sexual diversity, among other related themes, from a positive perspective based on human rights and diversity [43,45,58-60]. In general, this lack of education in diversity and sexual and gender minorities, highlighted by the health professionals interviewed, seems to be an obstacle to establishing an effective interaction regarding health interventions. This fact could impact the relationship between health professionals and PrEP users.

Though all the participants in this study, health professionals, and users did not face these problems, as these new forms of telehealth clinical monitoring are expanded to a larger population, in the case of PrEP users, challenges may arise, such as access to the internet, modern equipment that allows for more fluid communication, and the possibility of being connected to a network. These challenges should be seriously considered, especially for those with greater vulnerability. In addition, some users feel uncomfortable carrying out many remote consultations in a row, which suggests the need to maintain a certain degree of hybridity in preventive proposals

There are some limitations to be considered. The analyses presented are regarding users and professionals who chose to carry out clinical monitoring using TelePrEP. That is, we did not conduct qualitative interviews with users who decided to continue with in-person monitoring, and, consequently, we were unable to discuss the motivations that influenced the refusal of TelePrEP by almost half of the invited users [35] and for some professionals in the service who refused to collaborate in this component of the Combine! Study. Nonetheless, a structured questionnaire was applied after the moment of choosing between TelePrEP or in-person PrEP, and the analyses (unpublished data) reveal that motivations related to the excellent quality of the service and the possibility of resolving other health needs during medical consultations were frequent among those who chose to continue with in-person monitoring. This was particularly relevant for transgender women and travesties, who were included in the TelePrEP cohort but in a significantly lower proportion than expected. Most preferred in-person follow-up to address specific health needs, such as hormone therapy, underscoring the necessity of tailored care strategies for this population. Given these particularities, their data will be analyzed separately in a future publication.

Our research underscores the need for further exploration regarding teleconsultation and telemonitoring between health professionals and PrEP users. While our findings suggest that there are no extreme tensions in this new approach, it is crucial to understand the dynamics of this procedure in preventive medicine. We must be attentive to the ways in which the social actors involved adapt their practices and representations to this latest proposal. Thus, telemedicine as a proposal for health prevention care presents a series of complexities that require in-depth research to understand its scope and limitations in specific contexts and groups.

### Conclusions

In our study, we observed significant disagreement between users and health care professionals regarding telemedicine in PrEP services. While users perceive telehealth as a tool that increases autonomy, practicality, and convenience in HIV prevention, some health care professionals see it as a potential threat to the quality of care and the emotional bonds established during in-person consultations. Understanding the nature of these divergences in perceptions and assessments and how they imply the success of alternative forms of health care consists of essential efforts to ensure that the needs of all parties involved in the care process are adequately met. Nevertheless, it is vital to highlight that it is necessary to carry out new research of this type in contexts other than large Brazilian cities in order to contextualize different territories and socioeconomic realities to understand the underlying logic in the practices and representations of the implementation of a TelePrEP service.

Our study also contributes to further consolidating the evidence that points to the urgent need to rethink the health education curriculum so that health professionals have the necessary tools to provide comprehensive care to the most vulnerable populations, such as sexual and gender minorities. More specifically, our analyses also reveal the absence of these topics even in medical fields, as evidenced by statements from health care professionals, such as infectious diseases, which deal directly with taboos and socially stigmatized issues, such as sexual practices and risk.

This is the first and only study conducted in Brazil in which PrEP users were offered the possibility of undergoing clinical monitoring with non–face-to-face consultations even before the COVID-19 pandemic subsided in the country. Moreover, our findings will serve as a starting point for creating and improving telehealth services not only in HIV prevention but also in other strategic sectors by explaining motivations, perceptions, and experiences from the perspective of users and the professionals involved.

## Appendix

Multimedia Appendix 1 Interview scripts.

## Abbreviations

MSM: men who have sex with men
PrEP: pre-exposure prophylaxis
STI: sexually transmitted infection

## Data Availability

The data supporting the findings of this study are not publicly available due to privacy and confidentiality. However, deidentified data may be made available upon reasonable request to the corresponding author, subject to ethics approval and adherence to data use agreements.
