# Supplementary material for: Agreements and Disagreements Between Professionals and Users About the Experience of a Telehealth Service for HIV Pre-Exposure Prophylaxis (TelePrEP): Qualitative Interview Study
Source: J Med Internet Res. 2025 Apr 2;27:e67445. doi: 10.2196/67445 (PMC12004024; doi:10.2196/67445)
Supplement: Multimedia Appendix 1 [file jmir_v27i1e67445_app1.docx]

**Professionals**

**Introduction**

1. What is your full name?
2. How old are you?
   *(What is your date of birth: day, month, and year?)*
3. What is your skin color?
   *(If the person mentions another color)* Considering the five categories used by IBGE – white, black, brown, yellow, and indigenous – which best represents you?
   - Ask about being Black, exploring race/ethnicity and self-perception.
4. Do you follow or practice any religion? What is your spirituality like?
   *(If applicable)* Would you say religiosity is important or influences your life?
5. How do you identify in terms of your gender?
   *(If necessary, help clarify the concept of gender to ensure an accurate response.)*
   - Explain: not tied to biology; fluid, social, cultural, dialogical.
6. How do you identify in terms of your sexual orientation?
7. Could you briefly tell me about your academic journey? Have you pursued any further courses?

**Professionals’ Section**

1. Could you share a bit about your professional trajectory?
   - Focus on time working in the service and with PrEP.
2. What does PrEP represent in your daily professional routine, and what are the main challenges of working with it?
   - Perception of PrEP as a prevention strategy.
   - Challenges of integrating PrEP into routine work.
   - Differences between working with PrEP and other prevention strategies.
3. How do you perceive the relationship that users have with PrEP, the service, and the professionals who work here?
   - Values attributed by users to PrEP, the service, and the professionals.
4. To facilitate adherence and increase the number of PrEP users, discussions often highlight reducing service visits and the number of tests. How do you assess the impact of these reductions on the success of PrEP as a prevention strategy?
   - Impact on adherence.
   - Impact on the diagnosis of STIs.
5. How has your experience been with monitoring PrEP both in-person and remotely? What are the advantages and disadvantages of each modality?
   - Impact/changes on your professional routine.
   - Comparison of the modalities (in-person vs. remote) as prevention methods.
   - Professional gains and challenges with telePrEP.
   - Separate advantages and disadvantages for you as a professional, and for the users and the service.
6. TelePrEP raises concerns such as loss of connection with users, difficulty assessing their needs, worse adherence, or STI outcomes. Based on your experience with telePrEP, do you think these concerns have been confirmed?
   - How did you address these issues (loss of connection, decreased adherence, etc.)?
7. Based on your experience with telePrEP, what challenges have you identified?
   - Nature of the challenges (technical-operational, user/service adaptation).
   - Explore whether the challenges are the same as they were initially or if their nature/complexity has evolved.
   - Do challenges/problems persist?
   - Impact of these challenges on adherence and PrEP as a prevention strategy.
   - Strategies used to overcome these challenges.
8. What do you think is essential for telePrEP to work effectively?
   - What recommendations would you give to a service implementing telePrEP?
   - And what advice would you give to a professional starting to work with telePrEP?
9. What is your opinion on extending telePrEP to all PrEP users and all services? What might happen?
   - Is telePrEP suitable for all users?
   - Explore whether some users are perceived as less likely to adapt to telePrEP.

**About the Platform for Professionals**

1. Have you used the platform developed for telePrEP monitoring? Did it meet your needs?
   *(If applicable)* What needs (for professionals or users) could not be addressed through the platform?
2. What is your evaluation of the login screen? Do you suggest any improvements?
3. What is your evaluation of the remote medical assessment form? How could it be improved?
4. What is your evaluation of the "exams" section? How could it be improved?
5. What is your evaluation of the section where you prescribe PrEP? How could it be improved?
6. Did you access the informational materials available on the platform? What other materials or resources would you include in this section?
7. Did you interact with any users through the platform’s chat feature? How was that experience?
8. Would you suggest any modifications or improvements to the platform?

**Users**

**Introduction**

1. What is your full name?
2. How old are you?
   *(What is your date of birth: day, month, and year?)*
3. What is your skin color?
   *(If the person mentions another color)* Considering the five categories used by IBGE – white, black, brown, yellow, and indigenous – which best represents you?
   - Ask about being Black, exploring race/ethnicity and self-perception.
4. Do you follow or practice any religion? What is your spirituality like?
   *(If applicable)* Would you say religiosity is important or influences your life?
5. How do you identify in terms of your gender?
   *(If necessary, help clarify the concept of gender to ensure an accurate response.)*
   - Explain: not tied to biology; fluid, social, cultural, dialogical.
6. How do you identify in terms of your sexual orientation?
   *(If necessary, help clarify the concept to ensure an accurate response.)*
7. What is the highest level of education you completed? Have you pursued any further courses?
   - Explore formal and informal education levels.
8. Tell me a little about where and with whom you live.
   - Explore: housing conditions, neighborhood, community, household composition, and occupation, including personal income.
9. What is your occupation, or what kind of work do you do currently?
   - Investigate before and after the pandemic.
10. Is your job meeting your financial needs? Do you depend financially on someone, or does someone depend on you?
    - Explore the current economic situation and potential social vulnerability.
11. Have you ever engaged in sex work or received something in exchange for sex (such as gifts, favors, or privileges)?
    - Investigate all forms of transactional sex.

**About TelePrEP**

1. Tell me how you started using PrEP. Why did you choose this method?
   - Explore: How they learned about the method, in what context.
2. What was your life like (both general and in terms of your emotional/sexual dimension) when you started using PrEP? Why did you decide on PrEP?
   - Focus less on why they chose PrEP over other methods and more on potential combinations of methods.
   - How was your experience going to the service for PrEP follow-ups?
     - Explore: Relationship between users and health professionals (conflict? Trust, partnership?).
       *(If there are no mentions of benefits in going to the service, explore further.)*
   - When you had exclusively in-person consultations, did you ever pause or discontinue PrEP for any reason? If so, why?
     - Also explore the user's relationship with the service.

**Why did you choose TelePrEP? Tell me about your experience.**

- Explore: What were your expectations when you started TelePrEP, and have these expectations been met during your follow-up?
- How has your relationship with the health service and professionals changed since starting TelePrEP?
  - Explore visits to the service beyond picking up medications or routine lab tests.
- Has TelePrEP changed anything in your sexual life?
  - Explore: Changes in sexual practices; changes in the profile and number of partners; changes in PrEP usage and other prevention methods, etc.

**About the Platform**

- Did you use the platform developed for TelePrEP follow-up? Did it meet your needs?
  *(If applicable)* What needs were you unable to address through the platform?
- What is your evaluation of the login screen? Do you suggest any improvements?
- What is your evaluation of the remote medical assessment form? How could it be improved?
- What is your evaluation of the "exams" section? How could it be improved?
- What is your evaluation of the section where you retrieve your PrEP prescription? How could it be improved?
- Did you access the informational materials available on the platform? What other materials or resources would you include in this section?
- Did you interact with any professional through the chat feature on the platform? How was that experience?
- What other modifications or improvements would you suggest for the platform?
